# Validation of the Spanish version of the eating disorders quality of life instrument (EDQOL)

**DOI:** 10.1186/s40337-023-00832-w

**Published:** 2023-06-28

**Authors:** Yolanda Quiles Marcos, Álvaro Ruiz Maciá, Javier Manchón López, Eva María León Zarceño, María José Quiles Sebastián, María Roncero Sanchís, Maite España Alustiza, Pilar Arribas Saiz

**Affiliations:** 1 Department of Behavioral Sciences and Health, University Miguel Hernández, Avda. de la Universidad s/n, C. P. 03202 Elche, Alicante, Spain; 2 CREA: Center Specialized in the Treatment of Eating Disorders, University Miguel Hernandez Business- Science Park, Elche, Spain; 3 Department of Personality, Assessment and Psychological Treatments, University of Valencia, Valencia, Spain; 4 Department of Personality, Assessment and Psychological Treatments, University of Murcia, Murcia, Spain; 5 Unit of Eating Disordes, University Hospital de La Fe, Valencia, Spain

**Keywords:** Eating disorders, Quality of life, Validation, Psychometrics, EDQOL

## Abstract

**Background:**

The Eating Disorders Quality of Life instrument (EDQOL) is a disease-specific health related quality of life self-report questionnaire designed for disordered eating patients. Although the EDQOL is one of the most suitable and widely used questionnaires in many countries, no prior research has addressed the psychometric properties of the Spanish adaptation of the EDQOL. Therefore, the aim of this study is to examine the psychometric properties of the Spanish version of the EDQOL among ED patients.

**Methods:**

141 female eating disorder patients, with a mean age of 18.06 years (SD = 6.31), completed the EDQL in addition to the Eating Disorder Examination Questionnaire (EDEQ), the Depression, Anxiety and Stress Scales (DASS-21), the Clinical Impairment Assessment (CIA 3.0) and the Health Survey (SF-12). We calculated item/scale characteristics, internal consistencies and bivariate correlations with other measures of quality of life and adjustments. We assessed the goodness-of-fit of the 4-factor model using confirmatory factors analysis and explored the sensitivity of change following skill-based interventions.

**Results:**

The fit of the 4-factor model was acceptable (Root Mean Square Error of Approximation: 0.07, Standard Root Mean Square Residual: 0.07). Cronbach's alpha was excellent for the total (.91) and acceptable for all subscales (0.78–0.91). The construct validity was found with measures of psychological distress, depression, anxiety, quality of life and clinical impairment. The psychological and physical/cognitive scales and the EDQOL global scale were responsive to change.

**Conclusion:**

The Spanish EDQOL version is a useful instrument to assess quality of life in eating disorder patients and to evaluate outcomes of skills-based interventions.

## Abstract (plain language summary)

Eating Disorders (EDs) have a severe impact on many domains of quality of life (QOL). Therefore QOL needs to be addressed in effectiveness research and clinical practice. Furthermore, QOL is a very relevant concept in the treatment of chronic diseases and its evaluation requires specific health-related questionnaires. One widely used self-report measure to assess the quality of life in eating disorders is the Eating Disorder Quality of Life (EDQOL). Despite its high clinical relevance, EDQOL has not been previously investigated in the Spanish context. To overcome this gap, the present study aimed to validate the Spanish version of the EDQOL in a sample of Spanish eating disorder patients. The findings showed that the Spanish version of the EDQOL is robust in terms of reliability, factor structure and construct validity. This questionnaire may be preferred by clinicians and researchers interested in ED-specific health related quality of life impairment and as an additional indicator of ED severity.

## Background

Eating disorders (ED) have long been known to be associated with a wide range of impairment in physical and psychological domains, however patients who have suffered from an ED for a long time experience associated impairment in other important areas of daily life, such as in the work/study, family, social and leisure domains [1, 2]. This is the reason why, in the process of recovery in these patients, it is not only necessary to consider the “symptom improvement”, using domains such as frequency of ED behaviors (e.g., restricting, bingeing and purging), psychological symptoms, and general diagnostic severity, but it is also necessary to take into account how their lives are affected by such disorders in other important areas such as social, family and/or academic/occupational [3].

Literature review on Quality of Life (QOL) in EDs has shown that EDs are associated with more impaired QOL than those with a diagnosis of another psychiatric illnesses, including severe depression and healthy controls [1, 2, 4]. Despite the multitude of available quality of life instruments, concerns have been raised regarding the content validity of these instruments, and as a result, their suitability for use in mental health [5]. In the assessment of QOL two types of measures exist; one addressing a broad range of topics and indicated for use across conditions (generic measures), and another (disease-specific measures) focused on a certain condition or population. In the case of ED, criticisms have been made that generic measures may not be sensitive to the true level of impairment associated with EDs and may not capture the magnitude of disability caused by the illness or accurately differentiate between ED diagnostic groups [6, 7]. In contrast, specific questionnaires are more suitable at identifying the severity and response to treatment of the disease [3].

Five instruments have been developed to assess quality of life specifically in EDs: Eating Disorders Quality of Life (EDQOL) [6], Health-Related Quality of Life in Eating Disorders (HeRQoLED [8] and the short version HeRQoLED-s [9]), Eating Disorders Quality of Life Survey (EDQLS) [8] and Quality of Life Eating Disorders (QOLED) [11]. Tirico, Stefano and Blay [12] conducted a systematic review in which analyzed the characteristics of specific QOL instruments for eating disorders, and they concluded that the EDQOL, the HeRQoL and the EDQLS presented adequate development procedures and psychometric properties. Furthermore, a recent meta-analysis study carried out by van Krugten and colleagues [5] assessed the content validity and the suitability of existing QOL instruments for use in economic evaluations in mental health problems. This study concluded that among the specific instruments for assessing QOL in eating disorders, only the EDQOL included the seven dimensions, identified by Connell and colleagues, known to be important to the QOL of people with mental health problems [13, 14].

EDQOL is a disease-specific health related QOL self-report questionnaire designed for disordered eating patients [6]. It is a 25-item scale with four subscales (Psychological, Physical/Cognitive, Work/School, and Financial) and a meaningful total score. This questionnaire has good psychometric properties, EDOOL has shown high internal reliability that ranged from 0.86 to 0.95 and it has demonstrated adequate convergent and discriminant validity. An advantage of this questionnaire compared to others is that it includes only 25 items. It is well known that short instruments are more useful in epidemiological studies, clinical trials, and clinical practice, as short questionnaires improve compliance of patients and response rates and improve the quality of responses [15]. EDQOL may be useful as an outcome measure in clinical research, as a means of assessing patient improvement (or deterioration) in treatment. The development and validation study of the EDQOL showed that this instrument is sensitive to group differences between disordered eating and non-disordered eating groups, it differentiates groups based on symptom severity, it explains more symptom severity and group-related variance than a generic QOL instrument [6]. This questionnaire has shown excellent psychometric properties including adequate reliability and validity, in its Italian, Japanese and German versions [16–18].

To date, there are no studies that have adapted and validated the EDQOL in Spanish ED patients. The existing Spanish version of the HeRQoLED [8] and the short version HeRQoLED-s [9] have shown adequate psychometric properties and its reduced version facilitates the assessing of the QOL, but it has been criticized because it focuses predominantly on symptoms and behaviors [10] and doesn’t assess other important domains affected in ED such as work/school, financial or autonomy [5]. This is a preliminary study validation, and the aim was to examine the psychometric properties of the Spanish version of the EDQOL among ED patients.

## Method

### Participants

The sample consisted of 141 female participants from a clinical sample. The mean age was 18.06 (*SD* = 6.31, range 12–47). 56% (*n* = 79) of them met diagnostic criteria for Anorexia Nervosa restricting type (AN-R), 7.1% (*n* = 10) for Anorexia Nervosa purging type (AN-P), 11.3% (*n* = 16) for Bulimia Nervosa (BN), 5% (*n* = 7) for Binge Eating Disorder (BED) and 20.6% (*n* = 29 for Eating Disorder Not Otherwise Specified (EDNOS). The mean age of onset was 14.65 (*SD* = 2.93) and mean time of evolution was 44.42 (*SD* = 69.83) months. Their mean BMI was 19.73 (*SD* = 5.28). Regarding level of treatment, 36.9% (*n* = 52) of them received treatment in an ED specialized outpatient setting, 53.2% (*n* = 75) on a hospital-day and 9.9% (*n* = 14) in an inpatient unit. Regarding the level of education, 1.4% (*n* = 2) of them completed primary education, 72.4% (*n* = 102) secondary education, 11.3% (*n* = 16) superior education cycle and 14.9% (*n* = 21) university degrees.

### Procedure

Data was collected as part of the baseline assessment of research that evaluated a skills-based intervention for patients with an eating disorder (Trial Identifier: ISRCTN43554732). The Ethics and Research Integrity Committee of the university, as well as the hospitals where ED specialized units participated approved the conduct of this study.

The sample was collected in five different Spanish centers specialized in the treatment of ED. Once their informed consent was given, the participants completed the self-administered paper-and-pencil questionnaire. Afterwards, the therapists who attended the case provided the corresponding clinical data. No compensation of any kind was offered. To evaluate the sensivity to change of this questionnaire, we re-administered this scale another two months later following the completion of the skills-based intervention.

### Adaptation and cultural validation

The questionnaire’s translation and adaptation procedure took place using the guidelines for instrument translation across countries proposed by López-Roig and Pastor [19]: 1. Translation. Two bilingual people (residents in Spain whose native language was English) were first instructed about the study’s conceptual framework, and then they translated two versions into Spanish independently. This created the first Spanish version. 2. Back translation. The resulting version was translated back to English by two separate bilingual individuals who had not previously been informed about the objectives of the construct to be measured. The outcome was a version which is practically equal to the original. 3. Expert review. A team composed by members of the investigative group (two experts in eating disorders and one statistician) reviewed all versions and evaluated comprehension, as well as the semantic, linguistic, and conceptual equivalency. So after modifying and adjusting the instructions, and some items, a consensus was reached. 4. Pilot program. In order to evaluate the comprehension, reliability, and acceptance of both the items and the response scale, the questionnaire was administered to a pilot sample of 10 patients. The pilot sample was also interviewed, and opinions concerning different aspects related to understanding the instructions, the wording of the items, and so on were given. This resulted in some modifications being made to the Spanish version of the instrument.

### Instruments

*Sociodemographic and clinical items.*Age and educational level are reported by the patients. Clinical variables were completed by the therapist attending the case: diagnosis (according to the diagnostic criteria of the DSM-V), age of onset, time of evolution of the ED, treatment and BMI.

*Eating disorders quality of life (EDQOL)* [6]. EDQOL is a disease-specific health related QOL self-report questionnaire designed for disordered eating patients. It is a 25-item scale with four subscales (Psychological, Physical/Cognitive, Work/School, and Financial) and a meaningful total score. Participants respond to items on a scale from 0 (never) to 4 (always). EDOOL has shown high internal reliability that ranged from 0.86 to 0.95.

*Eating disorder examination questionnaire (EDEQ)* [20]. This scale measures the severity of psychopathology associated with eating disorder features. It consists of 36 items with a six-point Likert-type response scale distributed in four dimensions: restraint, eating concern, shape concern and weight concern. High scores indicate greater severity. The Spanish validation shows adequate internal consistency in the dimensions (α = 0.83, α = 0.75, α = 0.93, and α = 0.74, respectively) and in the global scale (α = 0.81) [21].

*Depression, anxiety and stress scales (DASS-21)* [22]. This scale measures emotional distress through 21 items rated on a four-point Likert-like scale distributed in three subscales of depression, anxiety, and stress. The Spanish validation shows adequate internal consistency in the subscales (α = 0.84, α = 0.70, and α = 0.82) [23].

*Clinical impairment assessment (CIA 3.0)* [24]. This scale measures psychosocial impairment due to ED features. It consists of 16 items rated on a four-point Likert-like scale distributed in three subscales of impairment: personal, social and cognitive. Higher scores indicate greater severity of clinical impairment. The Spanish validation has shown adequate internal consistency in the subscales (α = 0.92, α = 0.93, and α = 0.90, respectively) and in the global scale (α = 0.96) [25].

*Health survey (SF-12)* [26]. The SF-12 consists of a subset of 12 items from the Spanish validation of the SF-36. This scale measures health-related quality of life through 12 items rated on a three-to-four-point Likert-like scale. It is composed of eight scales to assess physical (general health, physical functioning, physical role and body pain) and mental health (vitality, social functioning, emotional role and mental health), which show adequate internal consistency (α = 0.85 and α = 0.78, respectively) [27].

### Data analysis

The statistical computing R environment 4.2.1 was used for the data analyses. The lavaan package [28] was used to conduct a Confirmatory Factor Analysis (CFA). The method of parameter estimation was MLR (maximum likelihood estimation with robust standard errors). According to Rhemtulla, Brosseau-Liard, and Savalei [29] the maximum likelihood method is suitable for variables with 5 or more categories and the sample size is small. The indices used for testing the model fit were the chi-square test, the comparative fit index (CFI > 0.90 indicates acceptable fit, > 0.95, good fit), the Tucker-Lewis index (TLI > 0.90 indicates acceptable fit, > 0.95, good fit), the root mean square error of approximation (RMSEA < 0.06), and the standardized root mean-square residual (SRMR < 0.08), following Hu & Bentler [30] criteria. These criteria, however, should be used with caution as the sample size is lower than N = 250, and maximum likelihood estimations tend to yield lower results in the CFI and TLI [30]. Particular attention was paid to the SRMR as it is a robust indicator regardless of the method of estimation [31]. In addition, the Akaike Information Criterion (AIC) was used to compare the fit of the models.

The psych package for R [32] was used to obtain the descriptive analyses, internal consistency (Cronbach’s α and McDonald’s ω coefficients), Pearson’s correlations, and Student’s t-test for paired samples.

## Results

### Factor structure

The CFA was carried out through the MLR estimation. Replicating the original study, a four-factor solution was tested, and 9 items were assigned to the psychological factor, 6 to physical/cognitive, 5 to economic, and 5 to work/school. Part of the results showed an acceptable fit of the model, according to the cutoff values proposed by Hu et al., [30] [χ2(269) = 440.09, *p* < 0.001; RMSEA = 0.07 (90% CI 0.06 ~ 0.08); SRMR = 0.07; CFI = 0.90]. One of the indices did not show an adequate fit (TLI = 0.88). The factors were correlated with each other.

An additional one-factor model was tested in order to compare the fit between the original and the single-factor model. In this case, the results showed that the fit of the one-factor model was not adequate (χ2(275) = 889.00, *p* < 0.001; RMSEA = 0.13 (90% CI 0.12 ~ 0.14); SRMR = 0.12; CFI = 0.61; TLI = 0.58). When AIC indexes were compared, the four-factor model was a more parsimonious solution (AIC = 9390.03) than the one-factor model (AIC = 10,140.48). Therefore, the original model was deemed the most appropriate. Parameter estimates of the four-factor model are presented in Table 1.

**Table 1:** Descriptive statistics of the items, item-factor correlations, CFA parameter estimates, and internal consistency

	*M (SD)*	Item-factor *r*	CFA parameter estimators	*α*	*ω*
*Psychological*				0.91	0.91
I1	3.64 (1.11)	0.66	0.69		
I2	4.04 (1.02)	0.70	0.73		
I3	3.17 (1.19)	0.72	0.76		
I4	3.55 (1.17)	0.66	0.70		
I5	3.31 (1.34)	0.71	0.76		
I6	3.56 (1.23)	0.73	0.78		
I7	3.60 (1.23)	0.63	0.67		
I8	3.29 (1.22)	0.73	0.76		
I9	3.13 (1.39)	0.60	0.64		
*Physical/cognitive*				0.85	0.86
I10	3.38 (1.41)	0.51	0.44		
I11	2.89 (1.32)	0.59	0.60		
I12	3.27 (1.27)	0.63	0.63		
I13	3.43 (1.21)	0.73	0.90		
I14	2.63 (1.35)	0.67	0.75		
I15	3.29 (1.25)	0.69	0.86		
*Financial*				0.79	0.83
I16	1.65 (1.12)	0.44	0.49		
I17	1.29 (0.82)	0.62	0.69		
I18	1.12 (0.51)	0.60	0.67		
I19	1.44 (0.96)	0.66	0.80		
I20	1.28 (0.77)	0.69	0.82		
*Work/academic*				0.78	0.80
I21	2.53 (1.57)	0.59	0.62		
I22	2.19 (1.25)	0.55	0.69		
I23	2.12 (1.44)	0.53	0.59		
I24	1.86 (1.47)	0.54	0.56		
I25	2.66 (1.34)	0.68	0.83		

*M (SD)* mean (standard deviation), *CFA* confirmatory factor analysis

### Reliability

Reliability coefficients for each of the factors are shown in Table 1. The global scale showed adequate internal consistency (*α* = 0.91; *ω* = 0.91).

### Construct validity

Eating pathology, emotional distress, psychosocial impairment, and quality of life measures were selected to examine its relations with ED related quality of life. The descriptive analyses of the selected variables, as well as the Pearson’s correlations with the EDQOL factors are shown in Table 2. Psychological and physical/cognitive factors correlated with all the variables. The financial factor correlated with all the variables except for the restraint scale of the EDEQ and the social impairment scale of the CIA 3.0. The work/academic factor correlated with all the variables except for the anxiety scale of the DASS-21.

**Table 2:** Descriptive analysis and correlations between EDQOL and other variables

	*M (SD)*	Range	Psychological	Physical/Cognitive	Financial	Work/Academy
EDQOL—Global score	2.56 (0.67)	1–5	0.79**	0.83**	0.56**	0.73**
Psychological	3.48 (0.92)	1–5		0.65**	0.28**	0.34**
Physical/cognitive	3.15 (0.99)	1–5	0.65**		0.31**	0.41**
Financial	1.36 (0.63)	1–5	0.28**	0.30**		0.28**
Work/academic	2.27 (1.05)	1–5	0.34**	0.41**	0.28**	
EDEQ—Global score	3.91 (1.36)	0–24	0.69**	0.57**	0.15	0.33**
Restraint	3.46 (1.79)	0–6	0.52**	0.52**	0.06	0.23**
Eating Concern	3.31 (1.33)	0–6	0.64**	0.54**	0.17	0.35**
Shape Concern	4.72 (1.41)	0–6	0.67**	0.49**	0.17*	0.31**
Weight Concern	4.17 (1.52)	0–6	0.67**	0.49**	0.19*	0.29**
DASS-21—Global score	33.31 (14.08)	0–63	0.64**	0.54**	0.25**	0.26**
Depression	12.55 (6.14)	0–21	0.63**	0.48**	0.20*	0.30**
Anxiety	8.59 (5.10)	0–21	0.53**	0.49**	0.23**	0.14
Stress	12.16 (4.83)	0–21	0.52**	0.46**	0.23**	0.22**
CIA3.0—Global score	28.29 (11.40)	0–48	0.75**	0.67**	0.25**	0.47**
Personal impairment	12.96 (4.63)	0–18	0.73**	0.52**	0.28**	0.28**
Social impairment	8.13 (4.50)	0–15	0.64**	0.60**	0.14	0.44**
Cognitive impairment	7.19 (3.86)	0–15	0.58**	0.65**	0.24**	0.55**
SF12—Physical Health	13.27 (3.19)	6–20	− 0.40**	− 0.43**	− 0.21*	− 0.39**
SF12—Mental Health	13.92 (3.94)	6–27	− 0.64**	− 0.51**	− 0.22*	− 0.31**

*M (SD)* mean (standard deviation), *EDQOL* Eating Disorders Quality of Life, *EDEQ* Eating Disorders Examination Questionnaire, *DASS-21* Depression Anxiety Stress Scales, *CIA3.0* Clinical Impairment Assessment, *SF-12* Health survey

**p < 0.01; *p < 0.05

### Responsiveness to change

Fifty-eight cases participated in a specialized ED intervention for two months and were assessed twice over time. Analyses of group means at Time 1 (T1) and Time 2 (T2) and responsiveness to change are shown in Table 3. The psychological and physical/cognitive scales and the EDQOL global scale were responsive to change. Their mean values were significantly reduced at T2 and showed a moderate responsiveness ranging from − 0.39 to − 0.59.

**Table 3:** EDQOL responsiveness of group means to change

	T1 (*N* = 58)	T2 (*N* = 58)	*t*	*p*	*SRM*
	*M (SD)*	*M (SD)*			
EDQOL-Global Score	2.49 (0.62)	2.28 (0.66)	2.93	0.01	− 0.39
Psychological	3.41 (1.00)	3.04 (1.03)	3.53	< 0.01	− 0.46
Physical/cognitive	2.96 (0.97)	2.46 (1.00)	4.47	< 0.01	− 0.59
Financial	1.24 (0.55)	1.27 (0.36)	− 0.31	0.76	0.04
Work/academic	2.32(0.90)	2.35 (1.12)	− 0.20	0.84	0.03

*EDQOL* Eating Disorders Quality of Life, *SRM* Standardized response means

## Discussion

The main aim of the present study was to analyze the psychometric properties of the Spanish version of the EDQOL in a sample of ED patients. Results obtained with the CFA analysis with the original four-factor model showed acceptable indices (except for the TLI), and superior to the one-factor model. Therefore, it was decided to keep the original four factors with all the items, as the saturations in all cases were adequate. Moreover, results at the level of internal consistency and construct validity were also satisfactory. As expected, the Spanish version of the EDQOL showed relationships with almost all the measures of eating pathology, emotional distress and psychosocial deterioration in the sense that the greater the eating symptoms, emotional distress and psychosocial deterioration, the worse the quality of life. This result has been found in previous studies using other versions of EDQOL [6, 16–18]. Specifically, our results posit that the psychological and physical/cognitive factors of the EDQOL scale were associated with all the variables. The financial EDQOL factor was related to all the ED symptomatology variables, apart from the EDEQ restriction scale and the CIA 3.0 social deterioration scale. Finally, the work/academic factor showed relationships with all the measured variables, except with the DASS-21 anxiety scale. The fact that the financial factor has a lower association when compared with the other measures could be due to the fact that the patients have an average age of 18 years old and are not able to have their own financial resources as they are not working. Furthermore, most of these patients were treated for ED in specialized public health centers.

Validation of the instrument in a Japanese sample has shown how the EDQOL subscales and the global quality of life score of patients with eating disorders correlate with most of the EAT-26 and EDI-2 subscales while they have not been found significant correlations between body dissatisfaction and the "physical/cognitive" and "work/school" subscales of the EDQOL [18]. The study by Mitchison et al. [33] found that all the EDQOL subscales, with the exception of financial, were significantly correlated with the symptomatology of the disorder assessed with the EDE. However, in our study, the economic factor is only related to two of the EDEQ symptom factors, specifically shape concern and weight concern. On the other hand, the trend of the EDQOL correlations with the SF-12 questionnaire carried out with a Spanish sample are similar to those found with a German sample and with the Italian version. Thus, the mental health factor of the SF-12 finds its highest degree of relationship with the psychological subscale of the EDQOL, followed by the physical subscale. As expected, in our study, the correlations with the greatest associations of the EDQOL with eating symptoms occurred with the psychological factor followed by the physical-cognitive factor. These results are also observed in similar studies that have evaluated eating symptoms with other instruments such as the EAT-26 and EDI [17, 18], which have found significant relationships among all factors [16].

On the other hand, the means obtained in our study show a medium level of quality of life (2.56 out of 5 in the overall score), with the psychological area presenting the worst level, followed by physical/cognitive and with the financial area as the best quality of life. This trend is also observed in other studies (Engel et al. 2006) and in the German and Japanese versions of the EDQOL [16, 17]. Regarding the responsiveness to change of the EDQOL, the results showed that the psychological, physical/cognitive scales, and the global EDQOL scales were sensitive to change when a second measurement was made after a skill-based intervention. This data would mean that these dimensions, precisely those in which there has been the greatest interference, would be useful as measures to assess the state of patients during treatment. However, other studies have found that the work/academic factor worked as a predictor at 6 and 12 months of follow-up after treatment, so this scale should be taken into account for possible changes [33].

QOL is a very relevant concept in the treatment of chronic diseases and its evaluation requires specific health-related questionnaires. This validation fills an important gap in the field of ED in Spain. This questionnaire may be preferred by clinicians and researchers interested in ED-specific HRQoL impairment and as an additional indicator of ED severity [33]. Therefore, it could be a useful instrument that allows patients to benefit from interventions directed at the areas most affected by ED.

Regarding its limitations, we must point out several relevant issues of this validation. First of all, we must highlight that, although the sample size is adequate, a limitation of this study is the small sample used to carry out a CFA of 33 items. Although the sample includes diagnoses of AN-P, AN-R, BED, and EDNOS, at least half of the sample corresponds to patients with a diagnosis of AN-R, which must be taken into account when interpreting the results. Therefore, more research is needed with the Spanish version in a larger sample and including a similar percentage of ED diagnoses.

Another limitation is regarding generalization to males. Although the prevalence of ED in females is higher than in males, it is important to include men in validation samples instruments in order to detect possible differences. Therefore, generalization of items to males should be used with caution. Something similar occurs with age, as the mean age of the patients was in their late teens. As the literature shows, it is to be expected that the impact on quality of life may vary with age and with the number of years of disease progression. This will have to be taken into account when applying this instrument to the adult population.

In relation to future psychometric analyses, it would be convenient to study temporal stability and measurement invariance (e.g., gender and diagnostic). Longitudinal studies should also be carried out in order to explore patient and treatment factors that may affect quality of life. This will allow for the development of specific interventions that target on these factors.

Finally, the information concerning a clinical sample of Spanish girls may not be generalizable to other Hispanic clinical samples, and further research is required to validate the factor structure in more diverse Hispanic groups. However, to our knowledge, this is the first study to assess the latent structure of the EDQOL among ED patients in a Hispanic population. Most research on ED has been conducted in populations from Western English-speaking countries [34]. Therefore, this study contributes to the development of cross-cultural research among Hispanics, in order to increase the understanding of ED among patients from understudied populations.

## Conclusions

The Spanish version of the EDQOL is an inexpensive, valid, and reliable instrument that assesses health-related quality of life specific to patients with EDs and is recommended for use both in research and clinical settings. Our study provides a useful tool to assess QOL among Spanish ED patients. Our Spanish version of the scale adds to the multitude of translated versions of this scale, allowing for cross-cultural comparisons of QOL among these patients.

## Data Availability

All data generated or analyzed during this study are not publicly available due the restrictions from the ethics committees.

## Appendix

Spanish translation of the EDQOL.

En los últimos 30 días …

**Table:**

nunca	Rara vez	A veces	A menudo	Siempre

### Psicológico

1. ¿Con qué frecuencia tu alimentación/peso ha dado lugar a que te sientas avergonzado/a o “diferente”?
2. ¿Con qué frecuencia tu alimentación/peso te ha hecho sentir peor contigo mismo/a?
3. ¿Con qué frecuencia tu alimentación/peso te ha hecho no querer estar con otras personas?
4. ¿Con qué frecuencia tu alimentación/peso te ha llevado a sentir que nunca te recuperarás?
5. ¿Con qué frecuencia tu alimentación/peso te ha hecho sentir solo/a?
6. ¿Con qué frecuencia tu alimentación/peso ha dado lugar a que tengas menos interés o placer en realizar actividades?
7. ¿Con qué frecuencia tu alimentación/peso te ha llevado a no cuidar de ti mismo/a?
8. ¿Con qué frecuencia tu alimentación/peso te ha hecho sentir raro/a, peculiar o extraño?
9. ¿Con qué frecuencia tu alimentación/peso te ha llevado a no comer delante de otras personas?

### Físico/Cognitivo

10. ¿Con qué frecuencia tu alimentación/peso te ha causado tener las manos o los pies fríos?
11. ¿Con qué frecuencia tu alimentación/peso te ha causado tener dolores de cabeza con frecuencia?
12. ¿Con qué frecuencia tu alimentación/peso te ha causado debilidad?
13. ¿Con qué frecuencia tu alimentación/peso ha afectado tu capacidad de prestar atención cuando querías?
14. ¿Con qué frecuencia tu alimentación/peso ha afectado tu capacidad de entender información verbal y escrita?
15. ¿Con qué frecuencia tu alimentación/peso ha reducido tu capacidad de concentrarte?

### Económico

16. ¿Con qué frecuencia tu alimentación/peso ha conducido a problemas con tu proveedor(es) de tratamiento en cuanto el coste del tratamiento?
17. ¿Con qué frecuencia tu alimentación/peso te ha llevado a tener problemas con pagar las facturas mensuales?
18. ¿Con qué frecuencia tu alimentación/peso te ha llevado a tener una deuda financiera significativa?
19. ¿Con qué frecuencia tu alimentación/peso te ha llevado a la necesidad de utilizar dinero de tus ahorros o utilizar tu tarjeta de crédito con frecuencia?
20. ¿Con qué frecuencia tu alimentación/peso ha dado lugar a la necesidad de pedir dinero prestado?

### Trabajo/Escuela

21. ¿Con qué frecuencia tu alimentación/peso ha conducido a una baja laboral?
22. ¿Con qué frecuencia tu alimentación/peso te ha llevado a sacar notas bajas?
23. ¿Con qué frecuencia tu alimentación/peso ha conducido a una reducción en las horas laborales en el trabajo?
24. ¿Con qué frecuencia tu alimentación/peso te ha llevado a perder tu puesto de trabajo o a abandonar los estudios?
25. ¿Con qué frecuencia tu alimentación/peso ha conducido a fallos en una clase o clases?

## Abbreviations

CFA: Confirmatory factor analysis
ED: Eating disorders
EDQOL: Eating disorder quality of life
HeRQoL: Health related quality of life
QOL: Quality of life
